# Pharmacokinetics and safety of rucaparib in patients with advanced solid tumors and hepatic impairment

**DOI:** 10.1007/s00280-021-04278-2

**Published:** 2021-04-28

**Authors:** Nikolay Grechko, Viera Skarbova, Monika Tomaszewska-Kiecana, Rodryg Ramlau, Piotr Centkowski, Yvette Drew, Rafal Dziadziuszko, Milada Zemanova, Jeri Beltman, Eileen Nash, Jenn Habeck, Mingxiang Liao, Jim Xiao

**Affiliations:** 1 Clinical Science, Clovis Oncology UK Ltd., Cambridge, UK; 2 Department of Internal Medicine and Clinical Pharmacology, Summit Clinical Research, Bratislava, Slovakia; 3 BioVirtus Centrum Medyczne, Jozefow, Poland; 4 Department of Oncology and Pulmonology, Poznan University of Medical Sciences, Poznan, Poland; 5 Department of Oncology, Provincial Specialist Hospital in Biała Podlaska, Biała Podlaska, Poland; 6 Clinical and Translational Institute, Newcastle University, Newcastle Upon Tyne, UK; 7 Department of Oncology and Radiotherapy and Early Clinical Trials Unit, Medical University of Gdańsk, Gdańsk, Poland; 8 Department of Oncology, First Faculty of Medicine, Charles University, Prague, Czech Republic; 9 Regulatory Affairs, Clovis Oncology, Inc., Boulder, CO USA; 10 Clinical Operations, Clovis Oncology, Inc., Boulder, CO USA; 11 Biostatistics, Clovis Oncology, Inc., Boulder, CO USA; 12 Clinical Pharmacology, Clovis Oncology, Inc., 5500 Flatiron Pkwy, Boulder, CO 80301 USA

**Keywords:** Rucaparib, Poly(ADP-ribose) polymerase inhibitors, Hepatic impairment, Safety, Pharmacokinetics

## Abstract

**Purpose:**

The poly(ADP-ribose) polymerase inhibitor rucaparib is approved for the treatment of patients with recurrent ovarian and metastatic castration-resistant prostate cancer; however, limited data are available on its use in patients with hepatic dysfunction. This study investigated whether hepatic impairment affects the pharmacokinetics, safety, and tolerability of rucaparib in patients with advanced solid tumors.

**Methods:**

Patients with normal hepatic function or moderate hepatic impairment according to the National Cancer Institute Organ Dysfunction Working Group (NCI-ODWG) criteria were enrolled and received a single oral dose of rucaparib 600 mg. Concentrations of rucaparib and its metabolite M324 in plasma and urine were measured. Pharmacokinetic parameters were compared between hepatic function groups, and safety and tolerability were assessed.

**Results:**

Sixteen patients were enrolled (*n* = 8 per group). Rucaparib maximum concentration (*C*_max_) was similar, while the area under the concentration–time curve from time 0 to infinity (AUC_0–inf_) was mildly higher in the moderate hepatic impairment group than in the normal control group (geometric mean ratio, 1.446 [90% CI 0.668–3.131]); similar trends were observed for M324. Eight (50%) patients experienced ≥ 1 treatment-emergent adverse event (TEAE); 2 had normal hepatic function and 6 had moderate hepatic impairment.

**Conclusion:**

Patients with moderate hepatic impairment showed mildly increased AUC_0–inf_ for rucaparib compared to patients with normal hepatic function. Although more patients with moderate hepatic impairment experienced TEAEs, only 2 TEAEs were considered treatment related. These results suggest no starting dose adjustment is necessary for patients with moderate hepatic impairment; however, close safety monitoring is warranted.

**Supplementary Information:**

The online version contains supplementary material available at 10.1007/s00280-021-04278-2.

## Introduction

Rucaparib is a potent, oral, small-molecule inhibitor of poly(ADP-ribose) polymerase (PARP) enzymes (including PARP1, PARP2, and PARP3) that exhibits activity against tumor cells with defects in DNA repair [1–5]. Rucaparib induces cytotoxicity in tumor cells with homologous recombination deficiency through a mechanism known as synthetic lethality, wherein enzymatic inhibition of PARP proteins in the presence of defects in the homologous recombination repair pathway (e.g., mutations in *BRCA1* or *BRCA2*) results in the accumulation of DNA damage and cell death [1, 4, 5]. Rucaparib is approved in the United States and European Union for treatment or maintenance treatment of patients with recurrent ovarian cancer and in the United States as single-agent therapy for patients with metastatic castration-resistant prostate cancer [6, 7].

The clinical pharmacokinetics (PK) of rucaparib have been well characterized among patients with advanced solid tumors. Rucaparib PK are described by a two-compartmental model with sequential zero- and first-order absorption and first-order elimination [8]. Across the dose range of 240–840 mg twice daily (BID), rucaparib displays linear PK with time-independent and dose-proportional increases in plasma exposure. At the 600-mg BID approved recommended dose, steady state is achieved after 1 week; the mean steady-state maximum plasma concentration (*C*_max,ss_) is 1940 ng/mL (54% coefficient of variation [CV]), and the area under the concentration–time curve from time 0 to 12 h (AUC_0–12 h_) is 16900 h⋅ng/mL (54% CV). Median time to steady-state maximum concentration (*t*_max,ss_) is 1.9 h. The mean absolute bioavailability of the rucaparib immediate-release tablet formulation is similar across doses, with a mean of 36% and a range of 30–45% [9]. The apparent steady-state clearance ranges from 15.3 to 79.2 L/h following rucaparib 600 mg BID administration [6, 10–12].

Population PK analysis was conducted based on available clinical data, which determined that interindividual variability was partially explained by baseline creatinine clearance (unpublished results). There was no apparent difference in PK between patients with normal or mildly impaired hepatic function [8]. However, there are limited data available from patients with hepatic impairment treated with rucaparib. In vitro, rucaparib has a low metabolic turnover rate [13]. Based on the results of a mass balance study, following a single oral dose of [^14^C]-rucaparib 600 mg, the mean terminal half-life (*t*_1/2_) is 25.9 h [11]. With 36% absolute oral bioavailability, as determined in a previous study, renal and hepatic elimination routes are estimated to represent ~ 30% and ~ 70% of total clearance, respectively [9, 11]. Rucaparib is metabolized via oxidation, N-demethylation, N-methylation, and glucuronidation. Seven metabolites have been identified (M309, M323, M324, M337a, M337b, M337c, and M500), with M324 as the most abundant metabolite [11]. In vitro PARP inhibition data demonstrate that M324 is not biologically active. However, because cytochrome P450 3A (CYP3A) and CYP1A2 mediate the formation of M324 [13], it is possible that hepatic impairment could affect rucaparib metabolism and M324 PK. The M324 PK data suggest that the formation of M324 accounts for ~ 1/3 of total clearance [11].

Here, we report results from Part 1 of a phase 1 trial designed to collect clinical PK and safety data to characterize the effect of hepatic impairment on rucaparib and its metabolite M324 in patients with advanced solid tumors.

## Materials and methods

### Study design and treatment

This was a 2-part, phase 1, open-label, parallel-group study (EudraCT 2017-001877-17). Part 1 evaluated the PK of rucaparib in patients with advanced solid tumors and moderate hepatic impairment compared with patients with normal hepatic function. Part 2 is an extension treatment phase for patients who participated in Part 1 and were eligible based on potential clinical benefit, as determined by the investigator. In Part 2, patients continued rucaparib treatment in 28-day cycles until progression of disease, unacceptable toxicity, death, loss to follow-up, withdrawal of consent, or other appropriate clinical reason for discontinuation. Results from Part 2 of the study will be reported separately.

In Part 1, patients received a single oral dose of rucaparib 600 mg on day 1; PK samples were collected and safety monitored through day 7. The primary study objective was to compare the PK parameters of a single dose of rucaparib in patients with advanced solid tumors and normal hepatic function to those in patients with advanced solid tumors and moderate hepatic impairment based on the National Cancer Institute Organ Dysfunction Working Group (NCI-ODWG) criteria for hepatic dysfunction (total bilirubin > 1.5× and ≤ 3× upper limit of normal with any level of aspartate aminotransferase) [14]. The secondary objective was to evaluate the safety and tolerability of rucaparib in patients with normal hepatic function as compared with patients with moderate hepatic impairment based on the NCI-ODWG criteria. In addition, as an exploratory measure, the study compared PK parameters for patients classified based on Child–Pugh criteria for hepatic dysfunction [15, 16].

The study was approved by national and local institutional review boards and was performed in accordance with the Declaration of Helsinki and Good Clinical Practice Guidelines of the International Conference on Harmonization. Patients provided written informed consent before participation.

### Patient population

Eligible patients included those aged ≥ 18 years with a body mass index (BMI) of 18.0–35.0 kg/m^2^ who had a histologically or cytologically confirmed advanced solid tumor determined by the investigator to potentially benefit from treatment with rucaparib. Patients had an Eastern Cooperative Oncology Group performance status (ECOG PS) of 0, 1, or 2 and adequate bone marrow and renal function.

Key exclusion criteria included anticancer treatment (chemotherapy, radiation, or other targeted agents) within 14 days or five times the half-life of the drug administered; unresolved grade ≥ 2 adverse events from prior therapies (except conditions associated with underlying liver disease in patients in the moderate hepatic impairment group); prior treatment with a PARP inhibitor (unless the PARP inhibitor was not the latest treatment, and it was discontinued > 3 months prior to the first dose on the study); pre-existing duodenal stent and/or any gastrointestinal disorder or defect that could interfere with absorption of rucaparib; corrected QT interval using Fridericia's formula (QTcF) ≥ 480 ms; clinically significant arrythmias or electrocardiogram (ECG) abnormalities; and arterial or venous thrombosis, myocardial infarction, unstable angina, cardiac angioplasty, stenting, or uncontrolled hypertension within 3 months.

For the hepatic dysfunction group, patients with moderate hepatic impairment were included based on NCI-ODWG classification criteria (total bilirubin > 1.5× and ≤ 3× upper limit of normal with any level of aspartate aminotransferase) [14]. Patients were required to have stable hepatic impairment as determined by the investigator. Patients with a history of liver transplantation, advanced ascites, or ascites that required drainage and albumin supplementation as judged by the investigator were excluded. Patients with grade > 2 hepatic encephalopathy or with a degree of central nervous system impairment that the investigator considered sufficiently serious to interfere with informed consent or with the conduct, completion, or results of the trial were also excluded.

### Sample collection and PK assessments

Sample collection, processing, and shipping were conducted according to standardized protocols shared across study sites. Plasma samples were collected for PK analysis at the predose assessment and at 1, 2, 3, 4, 8, 12, 24, 48, 72, 96, 120, and 144 h postdose. A predose urine sample was collected less than 12 h prior to study drug administration. Total urine was collected 0–12 h and 12–24 h postdose. Total (bound and unbound) concentrations of rucaparib and its metabolite M324 in plasma and urine samples were determined by Q Squared Solutions BioSciences (Ithaca, NY, USA) using validated liquid chromatography mass spectrometry/mass spectrometry methods. Stable deuterium-labeled analytes were used as internal standards. The assay calibration ranges for plasma samples were 5–10,000 ng/mL for rucaparib and 1–1000 ng/mL for M324 with lower limits of quantification (LLOQ) of 5.00 and 1.00 ng/mL, respectively. For urine samples, the calibration range was 500–50,000 ng/mL for both rucaparib and M324, with a LLOQ of 500 ng/mL for both analytes. The precision was within 9.3% CV, and the accuracy was within ± 6.2%.

PK parameters were estimated from the concentration–time profiles. Actual elapsed time after dosing was used to estimate plasma PK parameters based on noncompartmental methods using Phoenix® WinNonlin® version 8.1.0 (Certara USA, Inc., Princeton, NJ, USA). The PK parameters determined for rucaparib from the plasma concentration–time data included maximum concentration (*C*_max_), area under the concentration–time curve from time 0 to the time of last quantifiable concentration (AUC_0–last_)_,_ AUC from time 0 to infinity (AUC_0–inf_), *t*_1/2_, time to maximum concentration (*t*_max_), apparent clearance (CL/F), apparent terminal phase volume of distribution (V_z_/F), cumulative amount excreted in urine from time 0 up to 24 h postdose (Ae_0–24_), and renal clearance (CL_R_). Exploratory PK parameters for M324 calculated based on plasma and urine concentration-time data were *C*_max_, AUC_0–last_, AUC_0–inf_, *t*_1/2_, *t*_max_, Ae_0–24_, and CL_R_, as data allowed.

Safety and tolerability assessments comprised adverse events, clinical laboratory parameters, vital signs, 12-lead ECG, physical examination, and ECOG PS. For part 1 of this study, symptom-associated physical examination and safety laboratory tests, including clinical chemistry, hematology, coagulation, and urinalysis, were performed on day 7 after administration of the first dose of the study medication; 12-lead ECGs were performed 2 and 4 h postdose. All safety assessments were performed at the end of the treatment visit. Treatment-emergent adverse events (TEAEs) were defined as adverse events with an onset date on or after the date of first dose of study treatment until 28 days after the last dose. Causality of treatment-emergent adverse events was evaluated by investigators based on clinical and scientific data for rucaparib, including previously observed and known reactions, temporal relationship of event to rucaparib administration, patient response on dechallenge and rechallenge with the drug, medical history and ongoing concomitant conditions, natural course of the disease, potential effects of concomitant medications and interactions with the study drug, and biological plausibility of the event based on mechanism of action of rucaparib.

### Sample size and statistical analysis

The sample size was based on regulatory guidance from the US Food and Drug Administration, which recommends the inclusion of at least eight patients in each subgroup [17]. No formal sample size calculations were performed, as no prior information was available regarding the variability of rucaparib PK in patients with moderate hepatic impairment. To the extent possible, patients with normal hepatic function were matched with respect to sex, age, and BMI with patients who had moderate hepatic impairment.

PK parameters were calculated by noncompartmental analysis, analyzed using descriptive statistics, and presented by hepatic functional status. Analysis of variance (ANOVA) was used to compare PK parameters (e.g., *C*_max_, AUC_0–last_, and AUC_0–inf_) between the normal hepatic function group and the moderate hepatic impairment group per NCI-ODWG criteria. PK values were log-transformed before analysis. Baseline covariates (e.g., creatinine clearance [CLcr], sex, age, BMI, and ECOG PS) were tested for any significant effect (*P* value < 0.05). Geometric least-squares means were used to calculate the ratios of the PK parameters in the hepatic impairment group to those in the control group, along with 90% confidence intervals (CIs). The Wilcoxon rank-sum test was used to compare *t*_max_ values between the two groups, and estimates of the median differences were determined, along with 90% CIs. In addition, the relationships between log-transformed PK parameters (AUC_0–last_, AUC_0–inf_, *C*_max_) for rucaparib and M324 and hepatic function parameters (bilirubin and aspartate aminotransferase [AST]) relevant to the classification of hepatic impairment according to NCI-ODWG were explored by a linear regression approach. Summary statistics of the PK parameters were repeated with groupings based on Child–Pugh criteria as an exploratory analysis.

## Results

### Patients

Patient recruitment was conducted across 8 sites in Poland, Slovakia, the Czech Republic, and the United Kingdom. A total of 16 patients, eight with normal hepatic function and eight with moderate hepatic impairment per NCI-ODWG criteria, were enrolled. All patients completed Part 1 of the study and were evaluable for both PK and safety. Baseline characteristics were generally well balanced between the two groups (Table 1). Five patients had colon cancer, three patients had pancreatic cancer, three patients had extrahepatic bile duct cancer, two patients had lung cancer, and one patient each had prostate cancer, multifocal neuroendocrine cancer, or hepatocellular carcinoma. All eight patients with normal hepatic function and six of eight patients with moderate hepatic impairment had prior systemic anticancer therapy. For additional exploratory analyses using Child–Pugh criteria, seven patients were categorized as having a mild hepatic impairment, seven patients had a moderate hepatic impairment, and two patients had severe hepatic impairment.

**Table 1:** Demographics and baseline characteristics

	Normal hepatic function^a^ (*n* = 8)	Moderate hepatic impairment^a^ (*n* = 8)	Overall (*N* = 16)
Child–Pugh classification, *n* (%)			
Grade A (mild)	6 (75.0)	1 (12.5)	7 (43.8)
Grade B (moderate)	2 (25.0)	5 (62.5)	7 (43.8)
Grade C (severe)	0	2 (25.0)	2 (12.5)
Age, median (range), years	64.5 (43–77)	63.5 (30–74)	64.0 (30–77)
Sex, *n* (%)			
Male	7 (87.5)	7 (87.5)	14 (87.5)
Female	1 (12.5)	1 (12.5)	2 (12.5)
Race, *n* (%)			
White	8 (100.0)	8 (100.0)	16 (100.0)
ECOG PS, *n* (%)			
0	2 (25.0)	1 (12.5)	3 (18.8)
1	4 (50.0)	7 (87.5)	11 (68.8)
2	2 (25.0)	0	2 (12.5)
BMI, median (range), kg/m^2^	27.4 (18.5–32.9)	24.6 (18.2–34.9)	26.1 (18.2–34.9)
CLcr, median (range), mL/min	95.7 (68.8–115.8)	105.0 (52.9–207.8)	95.9 (52.9–207.8)
Type of cancer, *n* (%)			
Colon	3 (37.5)	2 (25.0)	5 (31.3)
Extrahepatic bile duct	0	3 (37.5)	3 (18.8)
Pancreatic	2 (25.0)	1 (12.5)	3 (18.8)
Non-small cell lung	2 (25.0)	0	2 (12.5)
Prostate	1 (12.5)	0	1 (6.3)
Hepatocellular	0	1 (12.5)	1 (6.3)
Neuroendocrine	0	1 (12.5)	1 (6.3)

*BMI* body mass index, *CLcr* creatinine clearance, *ECOG PS* Eastern Cooperative Oncology Group performance status, *NCI-ODWG* National Cancer Institute-Organ Dysfunction Working Group

^a^Based on NCI-ODWG criteria

### Pharmacokinetics

#### Pharmacokinetics in plasma

**Fig. 1:**
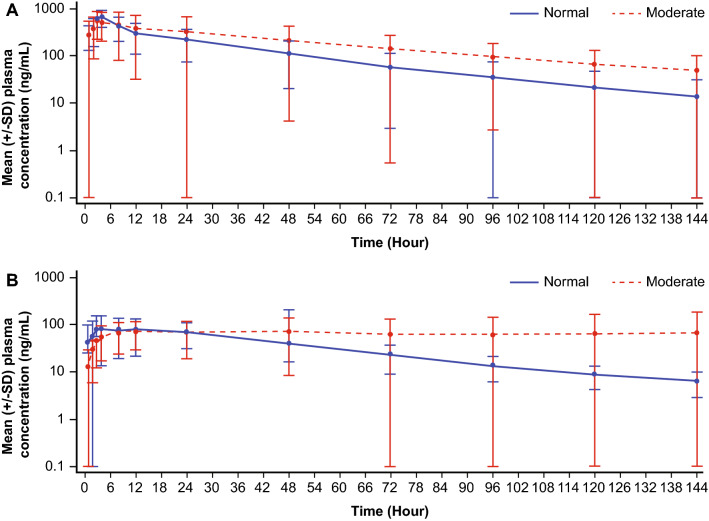
Mean (± SD) plasma concentration–time profile on a semi-log scale by hepatic function group (NCI-ODWG) for **a** rucaparib and **b** M324

Mean plasma concentration–time profiles by hepatic function groups based on NCI-ODWG criteria are shown in Fig. 1a, b for rucaparib and M324. The concentration–time plots show a steady decline of rucaparib concentration in plasma in both patient groups, with a slightly slower decline in the patient group with moderate hepatic impairment compared to the group with normal hepatic function. A steady decline of M324 plasma concentration was observed in the normal hepatic function group, but M324 levels remained high in the moderate hepatic impairment group.

The geometric mean (GM) *C*_max_ values for rucaparib in patients with moderate hepatic impairment and normal hepatic function were 583 ng/mL and 642 ng/mL, respectively. AUC_0–last_ was higher in patients with moderate hepatic impairment due to a lower GM apparent clearance (GM CL/F, 30.2 L/h) compared to patients with normal hepatic function (GM CL/F, 43.7 L/h). The GM half-life (*t*_1/2_) of rucaparib was longer in patients with moderate hepatic impairment (45.0 h vs 26.1 h in the normal hepatic function group; Table 2). PK parameters for M324 showed similar GM *C*_max_ values in patients with moderately impaired hepatic function and normal hepatic function, 76.7 ng/mL and 72.7 ng/mL, respectively. AUC_0–last_ was higher in patients with moderate hepatic impairment due to a longer GM *t*_1/2_ of M324 (58.1 h) compared to that of patients with normal hepatic function (37.1 h; Table 3). AUC_0–inf_ could not be calculated reliably for M324 due to an undefined terminal phase and/or high AUC extrapolation in most patients. Comparison of *C*_max_, AUC_0–last_, and AUC_0–inf_ parameters for rucaparib and M324 using ANOVA did not show any statistically significant differences between the moderate hepatic impairment and normal hepatic function groups classified by NCI-ODWG criteria (Table 4). Furthermore, the *t*_max_ values for rucaparib and M324 were highly variable and were not significantly delayed in the moderate hepatic impairment group compared to the normal hepatic function group. There was no apparent effect of hepatic impairment on the oral absorption of rucaparib and the formation kinetics of M324 based on *C*_max_ and *t*_max_ (Tables 2, 3 and 4).

**Table 2:** Summary of PK parameters of rucaparib

	NCI-ODWG		Child–Pugh		
	Normal hepatic function (*n* = 8)	Moderate hepatic impairment (*n* = 8)	Mild hepatic impairment (*n* = 7)	Moderate hepatic impairment (*n* = 7)	Severe hepatic impairment (*n* = 2)
*C*_max_, ng/mL					
PK, *n*	8	8	7	7	2
Mean (SD)	685 (249)	658 (358)	604 (288)	694 (240)	827 (641)
GM	642	583	550	657	692
Median (min–max)	665 (290–1150)	471 (325–1280)	590 (290–1150)	762 (420–1030)	827 (374–1280)
AUC_0–last_, h⋅ng/mL					
PK, *n*	8	8	7	7	2
Mean (SD)	16,200 (10,100)	25,500 (23,500)	11,600 (8170)	24,100 (9390)	42,200 (49,100)
GM	13,100	17,500	8910	22,500	23,900
Median (min–max)	17,700 (4390–34,600)	17,800 (3070–76,900)	8580 (3070–22,400)	18,400 (13,900–35,800)	42,200 (7460–76,900)
AUC_0–inf_, h⋅ng/mL					
PK, *n*	8	8	7	7	2
Mean (SD)	17,000 (10,900)	29,000 (26,900)	11,900 (8200)	26,800 (10,100)	48,300 (56,600)
GM	13,700	19,900	9370	25,300	27,100
Median (min–max)	18,100 (4500–37,900)	20,600 (3330–88,400)	8780 (3330–22,700)	21,100 (17,400–41,200)	48,300 (8290–88,400)
*t*_max_, h					
PK, *n*	8	8	7	7	2
Median (min–max)	3.97 (3.00–4.05)	3.96 (0.983–24.2)	3.98 (2.95–4.05)	3.95 (2.95–24.2)	4.49 (0.983–8.00)
*t*_1/2_, h					
PK, *n*	8	8	7	7	2
Mean (SD)	28.5 (12.5)	46.7 (12.6)	27.5 (12.6)	44.8 (14.6)	47.8 (1.07)
GM	26.1	45.0	25.2	42.6	47.8
Median (min–max)	26.3 (11.1–52.5)	49.3 (27.2–66.2)	26.6 (11.1–52.5)	49.9 (25.9–66.2)	47.8 (47.1–48.6)
CL/F, L/h					
PK, *n*	8	8	7	7	2
Mean (SD)	55.7 (42.7)	48.1 (56.8)	82.2 (59.5)	25.1 (8.62)	39.6 (46.4)
GM	43.7	30.2	64.0	23.7	22.2
Median (min–max)	33.1 (15.9–133)	29.1 (6.79–180)	68.3 (26.4–180)	28.4 (14.6–34.6)	39.6 (6.79–72.4)
V_z_/F, L					
PK, *n*	8	8	7	7	2
Mean (SD)	2220 (2380)	2840 (2500)	3340 (3160)	1660 (931)	2700 (3140)
GM	1650	1960	2320	1460	1530
Median (min–max)	1270 (876–7980)	2190 (476–7860)	2130 (876–7980)	1280 (725–3300)	2700 (476–4910)
Ae_0–24_, mg					
PK, *n*	8	7	7	6	2
Mean (SD)	25.4 (10.9)	19.8 (13.7)	20.7 (11.9)	29.8 (9.93)	8.89 (3.72)
GM	22.8	16.0	17.6	28.2	8.49
Median (min–max)	27.0 (7.69–40.6)	16.7 (6.26–41.5)	21.4 (7.69–40.6)	33.2 (16.7–41.5)	8.89 (6.26–11.5)
CL_R_, mL/min					
PK, *n*	8	7	7	6	2
Mean (SD)	53.5 (10.1)	46.2 (27.2)	56.7 (13.3)	52.3 (19.2)	20.1 (17.3)
GM	52.5	36.9	55.2	48.8	16
Median (min–max)	56.1 (33.5–63.6)	41.1 (7.89–76.7)	59.5 (33.5–76.7)	53.7 (22.9–73.2)	20.1 (7.89–32.3)

*Ae*_*0–24*_ cumulative amount excreted from time 0 up to 24 h postdose, *AUC*_*0–last*_ area under the concentration–time curve from time 0 to the time of last quantifiable concentration, *AUC*_*0–inf*_ area under the concentration–time curve from time 0 to infinity, *CL/F* apparent clearance, *CL*_*R*_ renal clearance, *C*_*max*_ maximum plasma concentration, *GM* geometric mean, *max* maximum, *min* minimum, *NCI-ODWG* National Cancer Institute-Organ Dysfunction Working Group, *PK* pharmacokinetics, *SD* standard deviation, *t*_*1/2*_ terminal phase half-life, *t*_*max*_ time to maximum plasma concentration, V_*z*_/F apparent volume of distribution during the terminal phase

**Table 3:** Summary of PK parameters of M324

	NCI-ODWG		Child–Pugh		
	Normal hepatic function (*n* = 8)	Moderate hepatic impairment (*n* = 8)	Mild hepatic impairment (*n* = 7)	Moderate hepatic impairment (*n* = 7)	Severe hepatic impairment (*n* = 2)
*C*_max_, ng/mL					
PK, *n*	8	8	7	7	2
Mean (SD)	90.1 (67.4)	105 (91.8)	111 (62.9)	64.1 (33.1)	164 (208)
GM	72.7	76.7	97.9	57.4	72.5
Median (min–max)	74.5 (28.9–235)	83.7 (16.9–311)	98.7 (42.7–235)	50.9 (28.9–118)	164 (16.9–311)
AUC_0–last_, h⋅ng/mL					
PK, *n*	8	8	7	7	2
Mean (SD)	4450 (2130)	8940 (9600)	5070 (1910)	5690 (4710)	15,900 (20,200)
GM	4000	5930	4740	4550	6840
Median (min–max)	3810 (1930–7890)	4760 (1550–30,200)	5130 (2330–7890)	3810 (1930–15,600)	15,900 (1550–30,200)
AUC_0–inf_, h⋅ng/mL					
PK, *n*	6^a^	3^a^	6^a^	3^a^	
Mean (SD)	5220 (2300)	4990 (720)	5800 (1720)	3840 (1590)	NC
GM	4750	4960	5590	3580	NC
Median (min–max)	4930 (2100–8340)	5230 (4190–5570)	5700 (3960–8340)	4190 (2100–5230)	NC
*t*_max_, h					
PK, *n*	8	8	7	7	2
Median (min–max)	10.1 (3.00–24.0)	30.0 (2.97–144)	4.00 (3.00–24.0)	23.9 (4.03–72.0)	73.5 (2.97–144)
*t*_1/2_, h					
PK, *n*	8	5^b^	7	6^b^	
Mean (SD)	39.3 (15.4)	65.6 (40.5)	35.8 (12.5)	65.3 (36.4)	NC
GM	37.1	58.1	34.4	58.9	NC
Median (min–max)	31.4 (25.8–64.7)	48.2 (38.9–135)	31.3 (25.8–62.7)	56.4 (38.0–135)	NC
Ae_0–24_, mg					
PK, *n*	8	7	7	6	2
Mean (SD)	13.7 (12.3)	5.46 (4.96)	16.1 (11.9)	5.44 (4.06)	1.38 (0.193)
GM	10.0	3.61	13.1	4.33	1.37
Median (min–max)	10.9 (3.47–40.9)	2.88 (1.24–12.8)	14.5 (4.77–40.9)	4.29 (1.46–12.8)	1.38 (1.24–1.51)
CL_R_, mL/min					
PK, *n*	8	7	7	6	2
Mean (SD)	120 (24.0)	72.7 (57.6)	121 (26.2)	90.5 (55.6)	39.3 (43.1)
GM	118	47.7	119	68.5	24.8
Median (min–max)	113 (96.7–161)	69.8 (8.84–163)	114 (94.7–161)	104 (12.7–163)	39.3 (8.84–69.8)

*Ae*_*0–24*_ cumulative amount excreted from time 0 up to 24 h postdose, *AUC*_*0–inf*_ area under the concentration–time curve from time 0 to infinity, *AUC*_*0–last*_ area under the concentration–time curve from time 0 to the time of last quantifiable concentration, *CL*_*R*_ renal clearance, *C*_*max*_ maximum plasma concentration, *GM* geometric mean, *max* maximum, *min* minimum, *NC* not calculated, *NCI-ODWG* National Cancer Institute-Organ Dysfunction Working Group, *PK* pharmacokinetics, *SD* standard deviation, *t*_*1/2*_ terminal phase half-life, *t*_*max*_ time to maximum plasma concentration

^a^Percent extrapolation ≤ 20% was required to retain AUC_0–inf_

^b^Percent extrapolation ≤ 20% and *r*^2^ > 0.80 was required to retain *t*_1/2_

**Table 4:** Statistical analysis (ANOVA) of rucaparib and M324 PK parameters and hepatic function (NCI-ODWG)

Analyte	PK parameters	Geometric LS means		Geometric mean ratio
		Moderate hepatic impairment (*n* = 8)	Normal hepatic function (*n* = 8)	Moderate/normal (90% CI)
Rucaparib	*C*_max_, ng/mL	583	642	0.907 (0.605–1.362)
	AUC_0–last_, h⋅ng/mL	17,500	13,100	1.335 (0.617–2.891)
	AUC_0-inf_, h⋅ng/mL	19,900	13,700	1.446 (0.668–3.131)
M324^a^	*C*_max_, ng/mL	76.7	72.7	1.055 (0.529–2.105)
	AUC_0–last_, h⋅ng/mL	5930	4000	1.483 (0.766–2.868)

*ANOVA* analysis of variance, *AUC*_*0–inf*_ area under the concentration–time curve from time 0 to infinity, *AUC*_*0–last*_ area under the concentration–time curve from time 0 to the time of last quantifiable concentration, *CI* confidence interval, *C*_*max*_ maximum plasma concentration, *LS* least square, *NCI-ODWG* National Cancer Institute-Organ Dysfunction Working Group, *PK* pharmacokinetics

^a^Statistical analysis of AUC_0–inf_ for M324 is not presented, as AUC_0–inf_ could only be calculated for three of eight patients with moderate hepatic impairment and six of eight patients with normal hepatic function

No statistically significant relationships were observed between PK parameters and baseline patient characteristics (CLcr, ECOG PS, sex, age, or BMI) in covariate analyses (*P* > 0.05). Linear regression of selected PK parameters (*C*_max_, AUC_0–last_, and AUC_0–inf_) for rucaparib and M324 and laboratory parameters (bilirubin and AST) relevant to the classification of hepatic impairment according to NCI-ODWG criteria also showed no statistically significant correlations for any of the parameters tested (*P* > 0.05; Table S1).

Similar trends were observed when patients were classified using Child–Pugh criteria for hepatic function. Mean plasma concentration–time profiles showed slower declines in rucaparib concentration and persistently high levels of M324 in patients with moderate or severe hepatic impairment compared to those with mild hepatic impairment (Fig. S1a, b), and AUC values increased in patients with increasing degrees of hepatic function (Tables 2 and 3).

#### Pharmacokinetics in urine

All patients had at least 24 h of total urine collection. Ae_0–24_ and CL_R_ estimates for rucaparib and M324 are reported in Tables 2 and 3, respectively. Despite comparable baseline estimated glomerular filtration rates between the two groups, CL_R_ for rucaparib was lower in patients with moderate hepatic impairment (GM, 36.9 mL/min) than in patients with normal hepatic function (GM, 52.5 mL/min). CL_R_ for M324 was also lower in patients with moderate hepatic impairment (GM, 47.7 mL/min) than in patients with normal hepatic function (GM, 118 mL/min). Similarly, the GM Ae_0–24_ of rucaparib and M324 excreted in urine was lower in the moderate hepatic impairment group than in the normal hepatic function group (Tables 2 and 3).

### Safety

A total of eight patients (50%) experienced at least 1 TEAE, including two patients (25.0%) in the normal hepatic function group and six patients (75.0%) in the moderate hepatic impairment group. The most commonly reported System Organ Class TEAEs were gastrointestinal disorders (three patients; 18.8%), including abdominal pain, ascites, and nausea (Table 5). Four patients (all in the moderate hepatic impairment group) experienced at least 1 TEAE of grade 3 or higher, including cholangitis, hyperbilirubinemia, hypokalemia, abdominal pain, and multiple organ dysfunction syndrome (one patient each). One patient in the moderate hepatic impairment group had grade 1 anemia and grade 1 thrombocytopenia that were considered treatment related as assessed by the investigator. After completion of PK assessment in Part 1, two patients subsequently withdrew from the study due to a TEAE (both in the moderate hepatic impairment group; one due to abdominal pain and one due to renal failure); both patients also experienced a serious adverse event (cholangitis and multiple organ dysfunction syndrome). The patient with multiple organ dysfunction syndrome subsequently died due to the event.

**Table 5:** Summary of any-grade TEAEs by preferred term

Summary of TEAEs, *n* (%)^a^	Normal hepatic function^b^ (*n* = 8)	Moderate hepatic impairment^b^ (*n* = 8)	Overall (*N* = 16)
Patients with any event	2 (25.0)	6 (75.0)	8 (50.0)
Abdominal pain^c^	0	1 (12.5)	1 (6.3)
Anemia	0	1 (12.5)	1 (6.3)
Ascites	0	1 (12.5)	1 (6.3)
Body temperature increased	0	1 (12.5)	1 (6.3)
Cholangitis^c^	0	1 (12.5)	1 (6.3)
Conjunctival hemorrhage	0	1 (12.5)	1 (6.3)
Decreased appetite	1 (12.5)	0	1 (6.3)
Hyperbilirubinemia^c^	0	1 (12.5)	1 (6.3)
Hypokalemia^c^	0	1 (12.5)	1 (6.3)
Multiple organ dysfunction syndrome^d^	0	1 (12.5)	1 (6.3)
Nausea	1 (12.5)	0	1 (6.3)
Pyrexia	0	1 (12.5)	1 (6.3)
Renal failure	0	1 (12.5)	1 (6.3)
Thrombocytopenia	0	1 (12.5)	1 (6.3)
Urinary tract infection	0	1 (12.5)	1 (6.3)

*TEAE* treatment-emergent adverse event

^a^All events were grade 1 or 2 unless otherwise noted

^b^Based on NCI-ODWG criteria

^c^Grade 3 event

^d^Grade 5 event

There were no clinically relevant treatment-related trends observed with respect to clinical laboratory parameters, vital signs, ECG, or physical examinations and hepatic dysfunction in patients treated with rucaparib.

## Discussion

Hepatic impairment can alter the exposure of a drug that is extensively cleared by the liver, resulting in changes in pharmacodynamics and increased drug-related adverse effects. Although in vitro metabolism studies and clinical results indicate that rucaparib has a low hepatic extraction ratio, the drug can be eliminated through multiple pathways, including CYP-mediated metabolism and renal and biliary excretion [6, 9, 11]. Formation of M324, the most abundant metabolite, is mediated by CYP3A and CYP1A2 [10, 11, 13]. This study was designed to assess the effect of hepatic impairment on the PK profile of rucaparib and M324 after a single dose of rucaparib 600 mg in patients with advanced solid tumors. Because rucaparib exhibits linear and time-independent PK, a single-dose design was selected to minimize uncertainty associated with dose deviation, concomitant medications, and food effects and allow for a more precise estimation of PK parameters for comparison between study groups [9, 10, 17]. The sample size of eight patients/group was aligned with recommendations from regulatory guidelines [17] and designs of similar studies [18–22].

The results from Part 1 of this trial suggest that moderate hepatic impairment, as defined by the NCI-ODWG criteria, has no apparent effect on the oral absorption of rucaparib, based on similar *C*_max_ and *t*_max_ values observed in both hepatic function groups. Patients with moderate hepatic impairment showed mildly higher AUC_0–inf_ and AUC_0–last_ values, consistent with slower hepatic elimination of rucaparib in these patients. However, the higher AUC values were likely confounded by slower renal clearance of rucaparib in patients with moderate hepatic impairment. For M324, the most abundant and biologically inactive metabolite of rucaparib, *C*_max_ was similar between the two groups, whereas AUC_0–last_ was higher in patients with moderate hepatic impairment. The *t*_max_ for M324 appeared to be highly variable and was numerically higher in the moderate hepatic impairment group than in the normal hepatic function group. Despite comparable CLcr baselines, CL_R_ values for rucaparib and M324 were lower in patients with moderate hepatic impairment compared to patients with normal hepatic function. Although the underlying reason remains unclear, this discrepancy may be related to transporter-mediated excretion, in addition to glomerular filtration of rucaparib and its metabolites.

For drugs with high plasma protein binding, unbound concentrations may affect PK parameters [23]. However, because rucaparib exhibits relatively low levels of plasma protein binding (70%) [6, 13], it is unlikely that decreases in plasma albumin in patients with hepatic impairment would significantly increase unbound concentration and, thereby, rucaparib clearance. Consistent with this assumption, population PK analysis testing did not identify baseline albumin levels as a clinically meaningful PK covariate [8]. Although M324 exhibits higher plasma protein binding (91%, unpublished results), in vitro target binding affinity and cytotoxicity assay results have demonstrated that the metabolite is inactive [7]. Based on these data, plasma binding was not monitored in this study.

Although patients with moderate hepatic impairment based on NCI-ODWG criteria showed mildly increased AUC_0–inf_ as compared to patients with normal hepatic function, overall, PK variability was moderate, and no statistically significant differences were observed for *C*_max_, AUC, or *t*_max_ for rucaparib and M324 between the groups. In addition, no relationship was observed between *C*_max_ and AUC for rucaparib and M324 and the baseline hepatic function parameters bilirubin and AST.

In this study, patients with moderate hepatic impairment assessed by NCI-ODWG criteria experienced more TEAEs and serious TEAEs with rucaparib, but only 2 TEAEs were considered treatment related by the investigators. No clinically relevant treatment-related trends were observed with respect to clinical laboratory parameters, vital signs, ECG, or physical examinations. However, safety data in Part 1 of this study are very limited, as only a single dose of rucaparib 600 mg was administered. Safety and tolerability of BID dosing in eligible patients are under investigation in Part 2, the optional extension phase of the study, and will be reported separately.

Although Child–Pugh criteria are often used for staging patients with hepatocellular carcinoma, it is not clear whether Child–Pugh scores correlate with the elimination of drugs metabolized by the liver [24–26]. For these reasons, the NCI-ODWG criteria were used as the primary measure of hepatic dysfunction in this study as they are tailored more for cancer patients. However, discordance between results from PK analyses using Child–Pugh and NCI-ODWG criteria have been observed, suggesting the importance of analyzing exposure using both criteria [27]. As an exploratory objective for the study, the PK parameters of rucaparib in patients with normal hepatic function were compared to those with hepatic dysfunction based on Child–Pugh criteria. Given the differences between the NCI-ODWG and Child–Pugh criteria, some patients fell into different categories of hepatic impairment depending on the criteria being used, although similar trends were observed when patients were classified based on either set of criteria. When Child–Pugh criteria were applied, two patients were characterized as having severe hepatic impairment; however, no conclusions can be drawn regarding severe hepatic impairment per Child–Pugh criteria due to the small sample size.

Previous clinical studies have demonstrated that rucaparib has a manageable safety profile with 600 mg BID as the starting dose [6]. The maximum tolerated dose of rucaparib was not reached with doses up to 840 mg BID in a dose escalation study [12]. In patients with ovarian cancer, *C*_max,ss_ was significantly correlated with several safety endpoints, but the correlations did not have predictive value, and no safety exposure *C*_max_ or AUC threshold could be clinically defined [28]. With respect to efficacy, exposure–response analysis of clinical data from the same studies revealed a trend between time-averaged steady-state AUC and independent radiologist reviewer-assessed objective response, suggesting that maximizing rucaparib dose may be associated with improved clinical efficacy. No correlation was observed for other efficacy endpoints, and no reliable surrogate or pharmacodynamics markers for a defined efficacy threshold were identified [28]. Based on these data, we suggest that rucaparib has a reasonable therapeutic range, and maximizing rucaparib dose while ensuring tolerability is an important clinical consideration in patients with moderate hepatic impairment.

Given the linear PK of rucaparib, the effect of hepatic impairment on rucaparib is likely similar after a single dose and at steady-state PK. The magnitude of increase in rucaparib AUC_0–inf_ associated with moderate hepatic impairment in this study is similar to changes in rucabarib AUCs observed as an effect of food and moderate renal impairment, which were not considered clinically significant and did not necessitate dose adjustment [6, 8, 10]. Thus, the results of this study also imply that the effects of moderate hepatic impairment on rucaparib PK is not be considered clinically significant, suggesting that no starting dose adjustment is necessary for patients with moderate hepatic impairment; however, patients with moderate hepatic impairment should be carefully monitored for hepatic function and adverse reactions.

## Supplementary Information

Below is the link to the electronic supplementary material.

Supplementary **Fig. S1** Mean (±SD) plasma concentration-time profile on a semi-log scale by hepatic function group (Child-Pugh) for (**a**) rucaparib and (**b**) M324 (PDF 71 KB)

Supplementary file2 (DOCX 42 KB)

## Data Availability

Requests for de-identified datasets for the results reported in this publication will be made available to qualified researchers following submission of a methodologically sound proposal to medinfo@clovisoncology.com. Data will be made available for such requests following the online publication of this article and for 1 year thereafter in compliance with applicable privacy laws, data protection, and requirements for consent and anonymization. Data will be provided by Clovis Oncology.
