# Supplementary figures and images for: Pharmacokinetics and safety of rucaparib in patients with advanced solid tumors and hepatic impairment
Source: Cancer Chemother Pharmacol. 2021 Apr 28;88(2):259–70. doi: 10.1007/s00280-021-04278-2 (PMC8236452; doi:10.1007/s00280-021-04278-2)

**Figure S1****A.**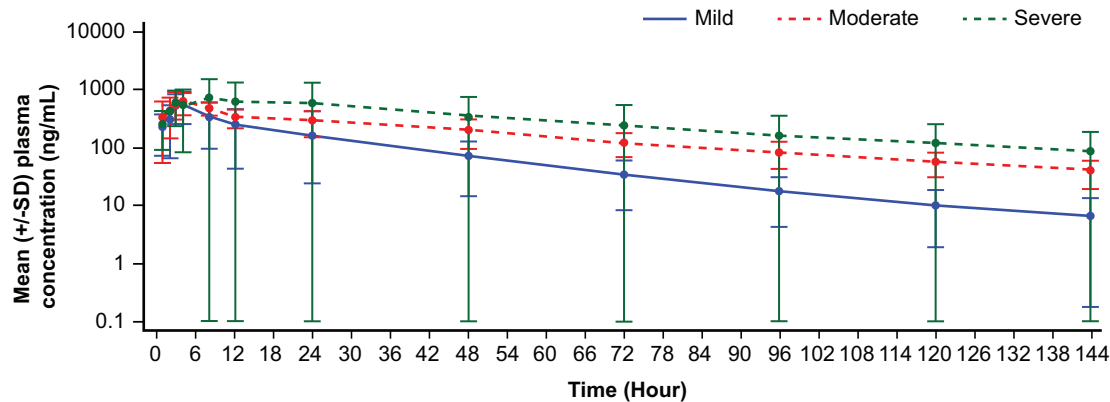**B.**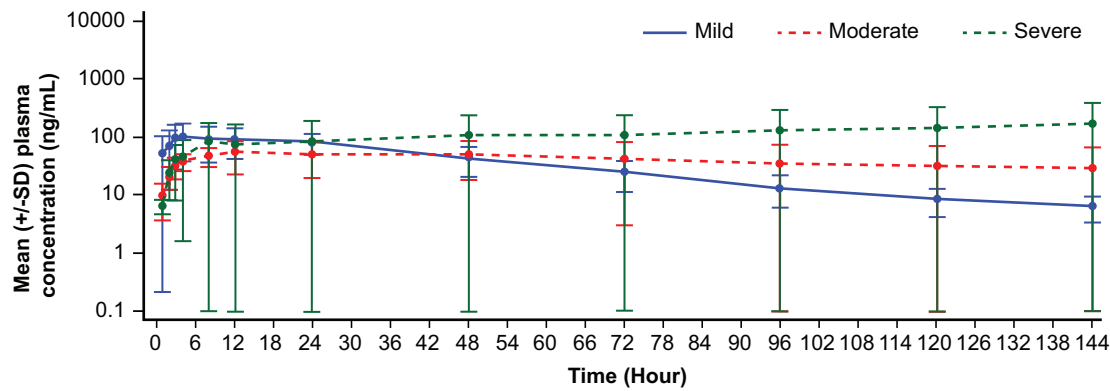

Supplement: Supplementary file 1 — Supplementary Fig. S1 Mean (±SD) plasma concentration-time profile on a semi-log scale by hepatic function group (Child-Pugh) for (a) rucaparib and (b) M324 (PDF 71 KB) [file 280_2021_4278_MOESM1_ESM.pdf]
