# Supplementary material for: Pharmacokinetics and safety of rucaparib in patients with advanced solid tumors and hepatic impairment
Source: Cancer Chemother Pharmacol. 2021 Apr 28;88(2):259–70. doi: 10.1007/s00280-021-04278-2 (PMC8236452; doi:10.1007/s00280-021-04278-2)
Supplement: Supplementary file 2 — Supplementary file2 (DOCX 42 KB) [file 280_2021_4278_MOESM2_ESM.docx]

# Table S1 Statistical analysis (linear regression) of pharmacokinetic and hepatic function parameters (NCI-ODWG)

| **Analyte** | **PK parameters** | **Hepatic function parameter** | **Slope estimate (90% CI)** | ***P* value** |
| --- | --- | --- | --- | --- |
| **Rucaparib** | C_max_ (ng/mL) | Bilirubin | –0.002 (–0.011 to 0.007) | 0.7395 |
|  |  | AST | 0 (–0.002 to 0.001) | 0.7140 |
|  | AUC_0-last_ (h*ng/mL) | Bilirubin | 0.002 (–0.015 to 0.019) | 0.8454 |
|  |  | AST | 0.001 (–0.002 to 0.004) | 0.6741 |
|  | AUC_0-inf_ (h*ng/mL) | Bilirubin | 0.003 (–0.014 to 0.020) | 0.7384 |
|  |  | AST | 0.001 (–0.002 to 0.004) | 0.6286 |
| **M324^a^** | C_max_ (ng/mL) | Bilirubin | –0.007 (–0.022 to 0.007) | 0.3821 |
|  |  | AST | 0 (–0.002 to 0.003) | 0.8215 |
|  | AUC_0-last_ (h*ng/mL) | Bilirubin | –0.004 (–0.018 to 0.011) | 0.6574 |
|  |  | AST | 0.001 (–0.001 to 0.004) | 0.3506 |

^a^Statistical analysis of AUC_0-inf_ for M324 is not presented, as AUC_0-inf_ could only be calculated for 3 of 8 patients with moderate hepatic impairment and for 6 of 8 patients with normal hepatic function.

AST, aspartate aminotransferase; AUC_0-inf_, area under the concentration-time curve from time 0 to infinity; AUC_0-last_, area under the concentration-time curve from time 0 to the time of last quantifiable concentration; CI, confidence interval; C_max_, maximum plasma concentration; NCI-ODWG, National Cancer Institute-Organ Dysfunction Working Group; PK, pharmacokinetics.
